# Development of a Spatio-temporal Contrast Sensitivity Test for Clinical Use

**DOI:** 10.18502/jovr.v17i1.10172

**Published:** 2022-01-21

**Authors:** Marcelo Fernandes Costa, Leonardo Dutra Henriques, Otávio Côrrea Pinho

**Affiliations:** ^1^Laboratório da Visão, Departamento de Psicologia Experimental, Instituto de Psicologia, Universidade de São Paulo, São Paulo, Brazil; ^2^Núcleo de Neurociências Aplicada, Universidade de São Paulo, São Paulo, Brazil

**Keywords:** Clinical Psychophysics, Drifting Grating, Dynamic Contrast Sensitivity, Primary Visual Pathway, Spatial Vision

## Abstract

**Purpose:**

We developed a contrast sensitivity test that considers an integrative approach of spatial and temporal frequencies to evaluate the psychophysical channels in processing two-dimensional stimulus for clinical use. Our new procedure provides a more efficient isolation of the magnocellular and parvocellular visual pathways supporting spatiotemporal contrast sensitivity processing.

**Methods:**

We evaluated 36 participants of both sexes aged 18–30 years with 20/20 or better best-corrected visual acuity. Two spatial frequencies (0.5 cycles per degree [cpd] and 10 cpd), being in one of the three temporal frequencies (0.5 cycle per second [cps], 7.5 cps, and 15 cps), were presented in a high-resolution gamma corrected monitor. A two-alternative forced-choice procedure was conducted, and the staircase method was used to calculate the contrast sensitivity. Reliability was assessed using a retest procedure within a month ( ± 5 days) under the same conditions.

**Results:**

Results showed statistical significance in 0.5 cpd and 10 cpd spatial frequencies for 0.5 cps (*F* = 77.36; *p* < 0.001), 7.5 cps (*F* = 778.37; *p* < 0.001), and 15 cps (*F* = 827.23; *p* < 0.001) with a very high (η ${}^{²}$ = 0.89) effect size. No statistical differences were found between the first and second sessions for all spatial frequencies. For reliability, a significantly high correlation and high internal consistency were found in all spatiotemporal conditions. The limits were calculated for normality.

**Conclusion:**

We developed an approach to investigate the spatiotemporal integration of contrast sensitivity designed for clinical purposes. The relative contribution of the low spatial frequencies/high temporal frequencies and the high spatial frequencies/low temporal frequencies of the psychophysical channels can also be evaluated separately.

##  INTRODUCTION

The evaluation of visual functions has improved in the last three decades with the development of visual tests for visual acuity (VA),^[1,2,3,4,5,6]^ contrast sensitivity (CS),^[7,8,9,10,11,12]^ color vision (CV),^[13,14,15,16]^ motion perception (MP),^[17,18,19,20]^ and stereopsis (ST),^[21,22,23,24,25,26]^ among others. Despite these developments and multiple studies showing that visual functions other than VA provide diagnosis for subclinical and early impairments in visual function,^[27,28,29,30,31,32,33,34,35,36,37,38]^ ophthalmology and visual sciences associated with optometry and orthoptic clinical practice have been preferentially using VA as a measurement of visual function.

The clear advantage of CS over VA measurements is the more detailed description of spatial vision, since symptomatic changes can occur in CS with VA within normal limits.^[39,40]^ Further, the test for VA is a measurement of the spatial separation function mediated by the parvocellular (PC) pathway, while CS measurements carry information mediated by the PC and magnocellular (MC) pathways.^[41,42,43,44,45]^ Since both pathways can be measured by a CS function, it is an obvious clinical test with more resources for diagnosis of visual impairments.

Clinical assessment of CS is mainly performed using charts such as Pelli-Robson and Functional Acuity Contrast Test (FACT).^[43,46]^ Both methodologies have significant limitations. The Pelli-Robson chart is based on a recognition VA test; however, it has a fixed low spatial frequency in the overall chart, and the contrast steps are based on three letters, which could lead to a learning effect after only a few uses. Another problem related to the Pelli-Robson chart is the need to read letters, which reduces testing potential for young children. The FACT is composed of five spatial frequencies and nine contrast levels. One important problem is that almost all participants with normal vision can see the last contrast level for middle frequencies, inserting a roof effect to reduce the sensitivity of the test. They were unable to see more than half of the contrast levels for low and high spatial frequencies. Furthermore, the suprathreshold contrast levels were also fixed, reducing the precision of sensitivity measurement. Despite these problems, these methods have been successfully used to measure CS in clinical settings.

Some studies have proposed alternative methods to isolate the contribution of the PC and MC pathways for psychophysical measurements of CS with relative success.^[47]^ However, they were designed to identify the psychophysical signature of the PC and MC pathways, and they took a long time to be completed (about one and a half hour), which made them unviable for clinical purposes.

Considering the above, we purposed a new CS test which intends to deal with chart measurement problems, and aimed to make the test user-friendly. The use of the same test for children and adults can make data comparable for development follow-ups, including a dynamic variable that amplifies the differences between PC and MC pathways. The possibility of isolating visual pathways is mandatory because many ocular and cerebral diseases affect these pathways differently. Our experience in the study of the traditional CS measurements in mercury,^[30,48,49,50]^ diabetes mellitus type 2,^[51,52]^ multiple sclerosis,^[53]^ and Leber's Ocular Hereditary Neuropathy^[54,55,56]^ motivated us to develop a CS test with greater efficiency in isolating PC and MC pathways for clinical testing.

##  METHODS

### Participants

We evaluated 36 participants (17 men and 19 women) with a best-corrected VA of 20/20 or better, measured using the ETDRS–Tumbling E chart (Xenônio Rep. Prod., São Paulo, Brazil). The exclusion criteria were absence of ophthalmologic complaints and/or diabetes mellitus, rheumatoid arthritis, systemic arterial hypertension, any other known systemic diseases, smokers, and the presence of dichromacy or anomalous trichromacy using the 38 plates version of the Ishihara pseudoisochromatic plates (Kanehara Trade Inc., Tokyo, Japan).

The participants, aged between 18 and 30 years (M = 22.6; SD = 3.7) were undergraduate and graduate students of the Institute of Psychology of the University of São Paulo. This study was approved by the local ethics committee via the approval number 66767317.5.0000.5561. All participants provided written consent for the inclusion of material about themselves and acknowledged that they could not be identified, as we ensured complete anonymity. The study followed the principles of the 1964 Declaration of Helsinki and its revised version.

### Equipment and Stimulus

CS functions were also measured psychophysically with the software PSYCHO for Windows v2.36 (Cambridge Research) using a Sony Trinitron 19 in. (GFD-420). The monitor was driven by a Cambridge Research VSG 2/4 graphics board with a refresh rate of 100 Hz non-interlaced and an 800 × 600 resolution.

The stimuli used were horizontal sinusoidal gratings with an average luminance of 10 fL, that is, 34.4 cd/m², measured using an Optical OP200-E photometer (Cambridge Research) and a visual angle of 4°. The luminance output of the screen was calibrated using a luminance meter (LS-110, Konica Minolta Sensing, Inc., Osaka, Japan). Screen uniformity was checked at maximum output. The contrast of the sinusoidal grating is defined as a Michelson contrast: 


C=L max −L min L max +L min ,

where *L ${}_{\max}$* is the maximum and *L ${}_{\min}$* is the minimum luminance consisting of a dimensional value. Gratings of 0.5 cycles per degree (cpd) and 10 cpd drifted rightward and leftward at temporal frequencies of 0.5, 7.5, and 15 cycles per second (cps). Testing was conducted in a dark room with the participants positioned 1 m away from the video monitor.

### Procedure

Participants were sat in a comfortable chair 1 m away from the monitor screen and were instructed to keep their eye fixed on a small black cross centered on the screen. Head stabilization was not performed. Ophthalmological patches (Oftan, AMP, São Paulo, Brazil) were used to cover one randomly chosen eye.

**Figure 1:**
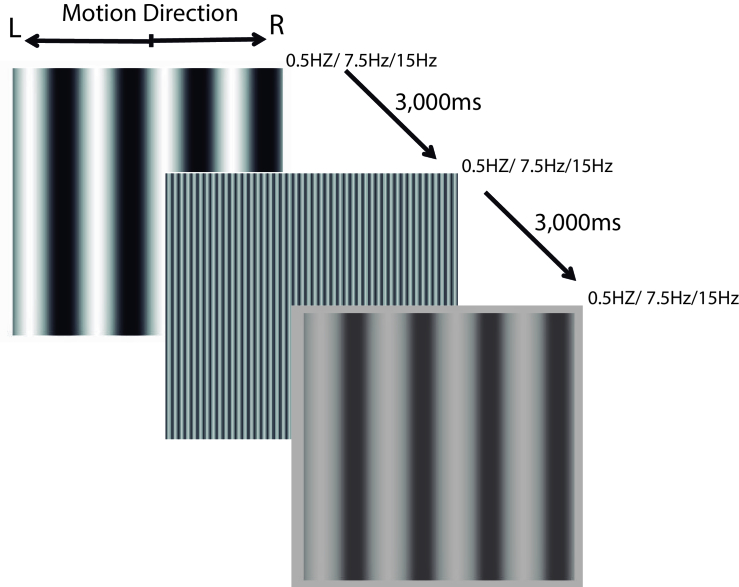
The illustrative timeline of the testing session. A spatial frequency randomly chosen was presented by 1 s, moving rightward or leftward in an also randomly chosen temporal frequency. The patient had up to 3 s to judge the movement of the grating in a two-AFC procedure.

At the beginning of the experiment, the participant was adapted to a gray mean luminance in the dark for 5 min. The stimulation consisted of a drifting grating with a randomly chosen spatial frequency presented by 1000 ms, followed by 3000 ms for the response in a two-alternative forced-choice (2-AFC) procedure, pressing a specific keyboard key for the right (m) and left (z) based on their perception of the grating's drifting side [Figure 1].

A psychophysical staircase procedure with a dynamic step size was used to determine the threshold. The staircase began with a high contrast level (70 ± 10 randomly chosen), which changed the luminance to the mean luminance background. The change depended on the participant's response: the grating contrast approached the background mean luminance every time there was a correct response and moved away from it when there was an incorrect response. The dynamic step consisted of a 50% reduction of the contrast level between the stimulus and background luminance. After the second reversal, the reduction changed to 12.5% between the stimulus level and background luminance. The contrast improvement always changed in increments of 25%. After seven staircase reversals, the program automatically calculated the contrast thresholds as the average luminance corresponding to the last five reversals. All testing procedures, including the adaptation time, lasted approximately 20 min.

For all spatial frequencies, the contrast thresholds were converted to CS according to the following equation: 


$$S=\frac{1}{Ct},$$

where *Ct* is the contrast threshold. To define the CS function, the CS for each spatial frequency was plotted.

Test reliability was estimated by comparing CS measurements in a test–retest design. The retest of the CS measurements was performed in all participants with a mean interval of one month ( ± 5 days) between the first and second measurements. The retests were also performed monocularly in the same eye and under the conditions of the first test.

### Statistical Analysis

Statistical analyses were performed using Statistica v.6.0.4, (StatSoft Inc., Tulsa, OK, USA, 2001). A complete descriptive analysis was performed. The normal distribution was checked by the Shapiro–Wilk and Kolmogorov–Smirnoff tests. A repeated-measures ANOVA was used to evaluate the statistical differences between spatial frequencies, drift velocity, and test–retest conditions. The correlation was calculated using the Pearson's product moment correlation test. No significant differences (*p* < 0.05) were observed between the conditions. The effect size, which is a quantitative measure of the magnitude of the experiment effect, was assessed using Cohen's *d* classification, that is, *d *= 0.2 was considered a “small” effect size, *d* = 0.5 represented a “medium” effect size, *d* = 0.8 a “large” effect size, and *d *= 1.4 a “huge effect” size.^[57]^ The interpretation of the effect size was that if the means of the two groups do not differ by 0.2 or more SD, the difference could be considered trivial, even if it is statistically significant.

##  RESULTS

All participants successfully completed both the first and second sessions. The CS measured in the first and second experimental sessions is described in detail in Table 1.

**Table 1:** Descriptive data from the first and second contrast sensitivity measurements


**Spatial frequency**	**First measurement**			**Second measurement**		
	**0.5 cps**	**7.5 cps**	**15 cps**	**0.5 cps**	**7.5 cps**	**15 cps**
0.5 cpd	174.2 (32.5)	332.6 (78.6)	163.2 (37.4)	174.8 (32,2)	332.6 (56.9)	167.2 (33.4)
10 cpd	113.7 (43.7)	76.2 (25.4)	34.7 (7.8)	117.7 (41.3)	77.7 (25.9)	35.2 (6.9)
	
cpd, cyle per degree; cps, cycle per second						

**Table 2:** Tolerance limits of contrast sensitivity for normality ranges


**Spatial frequency**	**Tolerance limits**		
	**0.5 cps**	**7.5 cps**	**15 cps**
0.5 cpd			
Upper	240.3	492.2	239.1
Lower	108.2	173	87.3
10 cpd			
Upper	202.5	127.9	50.5
Lower	24.8	24.6	18.8
cpd, cyle per degree; cps, cycle per second			

**Figure 2:**
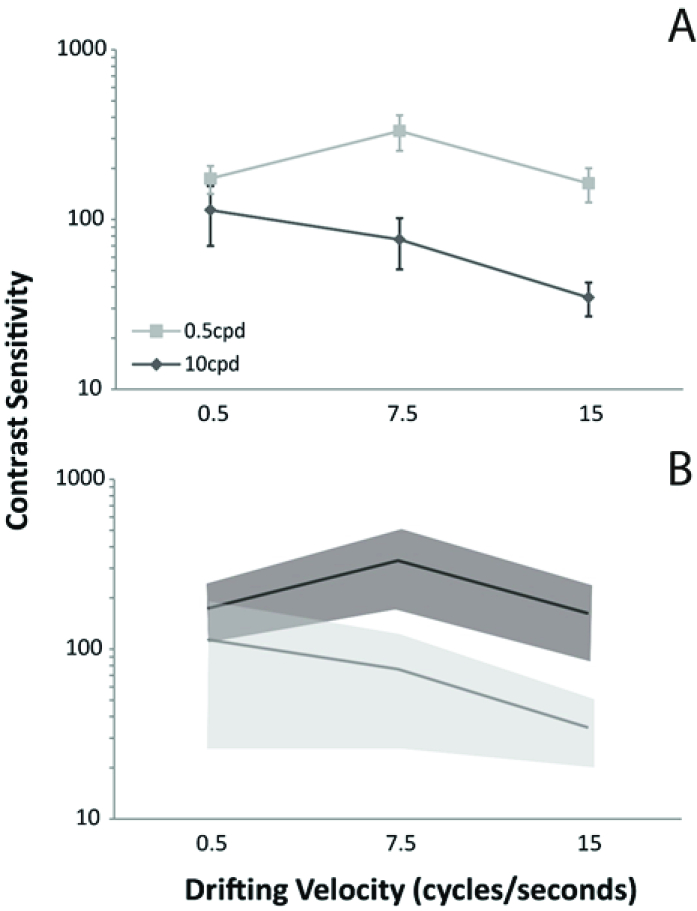
The psychophysical CS signature for the low (0.5 cpd) and high (10 cpd) spatial frequency. (A) The mean and standard deviation of the spatiotemporal interaction. (B) The normality range to be used for clinical purposes. In both panels, it is evident that the middle and high temporal frequencies are more discriminable areas to isolate the MC and PC contribution in the CS.

Considering the spatial frequencies of the grating, statistically significant results were found between 0.5 cpd and 10 cpd for 0.5 cps (*F* = 77.36; *p* < 0.001), 7.5 cps (*F* = 778.37; *p* < 0.001), and 15 cps (*F* = 827.23; *p* < 0.001) in the first and second sessions. The calculated effect size was considered to be very high (η ${}^{²}$ = 0.89). No statistical difference was found between the first and second sessions for all spatial frequencies for 0.5 cps (*F* = 0.11; *p* = 0.73), 7.5 cps (*F* = 0.06; *p* = 0.93), and 15 cps (*F* = 0.24; *p* = 0.63). The results are shown in Figure 2.

**Figure 3:**
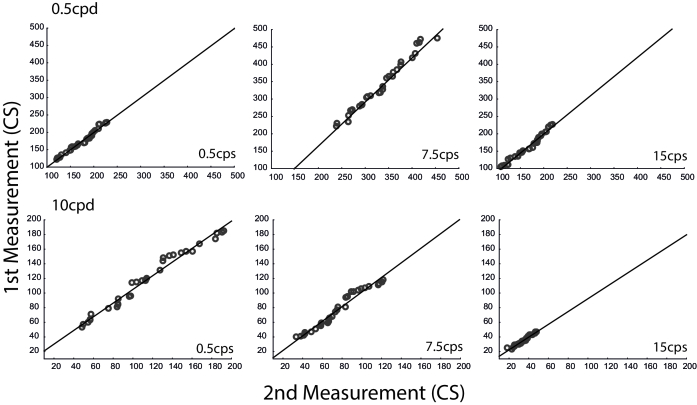
Correlations between the first and second measurements for reliability purposes. A strong correlation is evident for both low and high spatial frequencies in all temporal frequencies.

Reliability was assessed using Pearson's correlation, and the results are shown in Figure 3. A significantly high correlation was found in 0.5 cpd for 0.5 cps (*r* = 0.988; *p* < 0.001), 7.5 cps (*r* = 0.919; *p* < 0.001), and 15 cps (*r* = 0.985; *p* < 0.001), and in 10 cpd for 0.5 cps (*r* = 0.989; *p* < 0.001), 7.5 cps (*r* = 0.972; *p* < 0.001), and 15 cps (*r* = 0.980; *p* < 0.001).

Internal consistency was assessed using Cronbach's alpha coefficient. A high internal consistency was obtained for 0.5 cpd (α = 0.8449) with high item-total correlation for 0.5 cps (*r* = 0.880), 7.5 cps (*r* = 0.836), and 15 cps (*r* = 0.894). Similarly, a high item-total correlation was also observed for 10 cpd (α = 0.8963) to 0.5 cps (*r* = 0.997), 7.5 cps (*r* = 0.994), and 15 cps (*r *= 0.979).

Based on the tolerance limits, we calculated the boundaries of the CS normality range for clinical purposes. Tolerance limits were calculated considering the mean value (*X*) and with a factor (*k*) multiplied by the standard deviation (SD).^[58]^ The *k* factor can be chosen considering the percentage of the population covered (90%, 95%, or 99%) and the significance level of 0.90, 0.95, or 0.99, according to the number of participants. For our number of participants, we used the value 2.03, in which we covered 95% of the population with a *p*-value of 0.95. The tolerance limits are presented in the rightmost columns of Table 1.

##  DISCUSSION

Clinical evaluation of CS has been improved in ophthalmological clinics^[8,9]^ and it is a good step forward in understanding the spatial vision of their patients, as VA is a one-dimensional evaluation and CS is a two-dimensional test, since contrast is added to each spatial frequency measured. In our study, we included one more dimension by adding temporal modulation, which is a significant improvement, considering that objects in our visual environment are frequently moving. Using this more complex approach to investigate spatial vision, we can potentially be able to help our patients more efficiently and conduct more informative studies about visual and ophthalmological diseases and about the visual impairment suffered.

The robustness of our CS test was addressed by calculating the validity and reliability of the measurement. Validity was assessed by measuring the Pearson's correlation coefficients of the first and second measurements. We had a high correlation coefficient for both low (0.5 cpd) and high (10 cpd) spatial frequencies, regardless of the temporal frequency used. Since both the first and second measurements were highly correlated, this suggested that the influence of external variables had a low impact on the results obtained. Further, the reliability was considered high, suggesting that the reliability of our test was strong.

We also compared the mean contrast sensitivities of the first and second measurements. The absence of statistical significance in the same spatial frequencies compared to the first and second testing sessions and the statistical difference between the temporal frequencies within the first and the second testing sessions corroborate that our new CS test is robust.

An additional advantage of our measurement is the possibility of isolating the MC and PC visual pathways that contribute to CS. Using the amplitude of extracellular synaptic potential recordings for different Michelson contrast levels, retinal cells that project to the MC layer of the lateral geniculate nucleus (LGN) showed a logarithmic curve, in which huge improvements in amplitude responses occurred with small contrast increments. For the retinal ganglion cells projecting to the PC layer of the LGN, a linear curve was modeled with a small increase in amplitude response with a moderate increase in contrast levels.^[41]^ The psychophysical correlates of these visual pathways were obtained using a pedestal paradigm.^[47]^ MC-inferred responses were related to high temporal frequencies and PC-inferred responses related to low temporal frequencies. According to the spatial profile, low spatial frequencies were related to the MC-inferred pathway and the PC-inferred pathway related to high spatial frequencies.^[45]^ The results obtained in our test are in line with earlier studies since the high spatial frequency with a low temporal modulation had better CS than the results for the middle or high temporal frequencies. On the other hand, better CS was obtained for the middle and high temporal modulations for low spatial frequencies.

Furthermore, we found a significantly different signature of CS spatiotemporal integration. For the low spatial frequency, there was a reduction in CS as the temporal frequency increased in an almost linear fashion. For the high spatial frequency, the curve had an inverted U-shape, in which there was an increase in CS as the temporal frequency increased from 0.5 to 7.5 Hz and then, there was an inversion of the relation since the CS reduced as the temporal frequency increased from 7.5 to 15 Hz. The different signatures of the MC- and PC-CS measured psychophysically have two important implications. First, the test was successful in isolating the MC and PC contributions of the measured spatiotemporal CS. Second, the difference in the CS shape has an important contribution for diagnostic purposes, adding resolution to the CS measurement.

The isolation of MC and PC pathways has a huge clinical significance because many ophthalmological and neurological diseases affect these visual pathways differently. Visual impairment related to reading problems in children with learning difficulties and dyslexia has been related to the reduction of CS at low spatial frequencies, suggesting an MC pathway failure.^[32]^ MP, also an MC pathway function, is impaired in children with Down's Syndrome^[34]^ and strabismic amblyopia.^[28,59]^ We believe that our test improves the MC- and PC-mediated CS measurements because it integrates spatial and temporal proprieties in one measurement.

Considering the significant clinical applications, we calculated the normality range for clinical purposes. Of course, there are some restrictions on the use of normal ranges, as we calculated based on the age of our sample. For children and elderly patients, additional measurements should be performed in future studies.

In summary, we developed a test to investigate the spatiotemporal integration of CS, designed for clinical purposes. The relative contribution of the low spatial frequencies/high temporal frequencies, and the high spatial frequencies/low temporal frequencies of the psychophysical channels can also be evaluated separately. The validation and replicability were highly successful, and tolerance limits were calculated to define the normality ranges.

##  Financial Support and Sponsorship 

This research was supported by grants from FAPESP (Projeto Tematico # 2014/26818-2 to Dora Fix Ventura and Marcelo Fernandes Costa); CNPq #401153/2009-6 to M.F.C.; M.F.C. is CNPq research fellow.

##  Conflicts of Interest

The authors declare that they have no conflict of interest.
